# Supplementary material for: Investigating impacts of the mycothiazole chemotype as a chemical probe for the study of mitochondrial function and aging
Source: GeroScience. 2024 Apr 3;46(6):6009–28. doi: 10.1007/s11357-024-01144-w (PMC11493899; doi:10.1007/s11357-024-01144-w)
Supplement: Supplementary file 1 — (PDF 893 kb) [file 11357_2024_1144_MOESM1_ESM.pdf]

A.

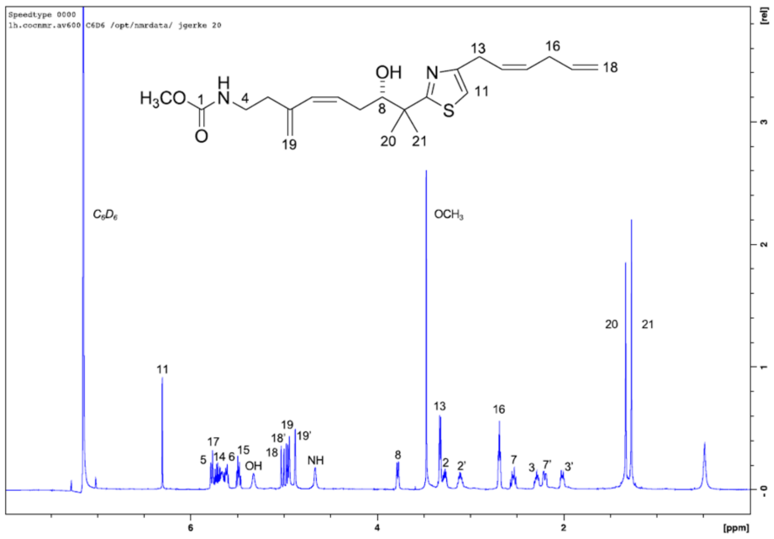

B.

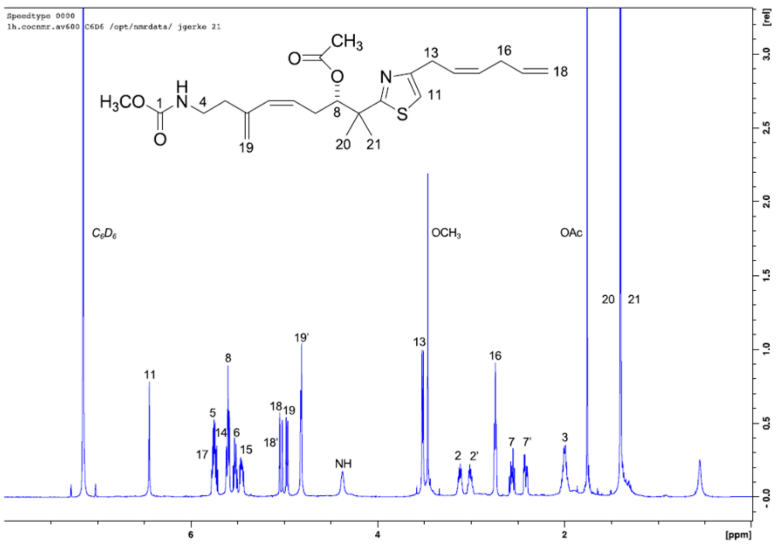

Fig: S1

Fig.s1. ^{1}H NMR analysis of (A) mycothiazole (MTZ) and (B) 8-*O*-acetylmycothiazole (8-OAc).

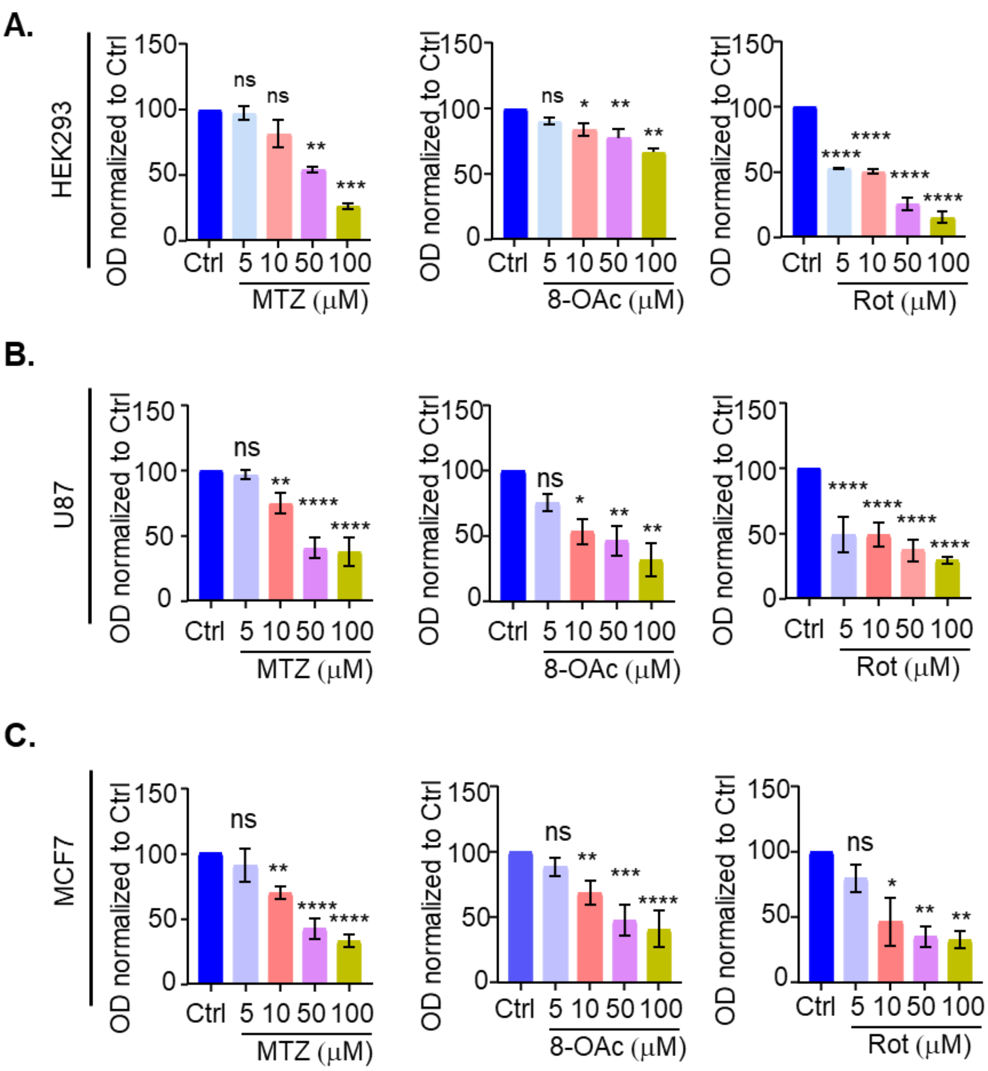

Compounds	IC50
MTZ	62.95
8-OAc	155.61
Rote	30.97

Compounds	IC50
MTZ	50.54
8-OAc	43.91
Rote	37.77

Compounds	IC50
MTZ	45.96
8-OAc	46.79
Rote	48.60

Fig: S2

Fig. s2. 8-OAc show selective toxicity towards cancer cells in comparison to MTZ and Rote.

Cytotoxicity of (A) HEK293T non-cancer cells (B) U87, human glioblastoma (C) MCF7 human breast cancer cells by MTT assay after treatment with vehicle/DMSO or indicated concentrations of MTZ, 8-OAc, and Rote treatment for 24h. Corresponding charts show the IC50 values of the small molecules for different cells. Data is presented as OD values normalized to the DMSO-treated control. Corresponding chart shows the IC50 values of cells. Bar graphs represent mean and standard deviation. All statistical analysis was performed by one-way ANOVA using GraphPad Prism 10. ns = not significant, * = p < 0.03; ** = p <0.002; *** = p<0.0002; **** = p< 0.0001.

A.

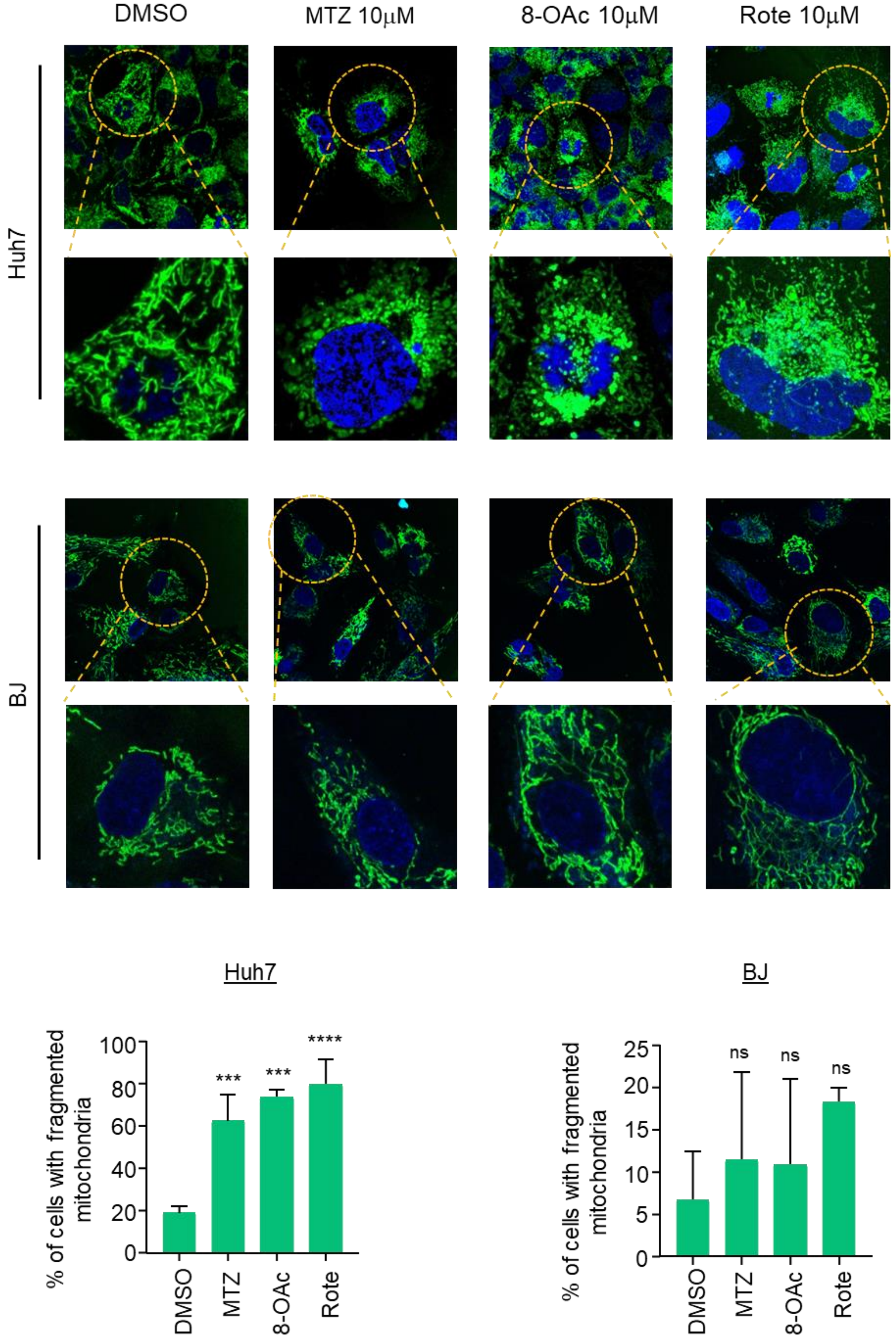
DMSO
MTZ 10μM
8-OAc 10μM
Rote 10μM
Huh7
BJ
Huh7
% of cells with fragmented mitochondria
100
80
60
40
20
0
***
***
****
DMSO
MTZ
8-OAc
Rote
BJ
% of cells with fragmented mitochondria
25
20
15
10
5
0
ns
ns
ns
DMSO
MTZ
8-OAc
Rote

Fig: S3

Fig. s3. MTZ, 8-OAc, and Rote preferentially cause mitochondrial fragmentation to cancer cells and not non-cancer cells.

(A) Representative images of Huh7 (top) and BJ fibroblast (bottom) cells treated with DMSO or 10 µM MTZ, 8OAc, and Rote treated with DAPI and mitotracker green as described in Materials and Methods. The corresponding bar graph shows the graphical representation of the percentage of cells with fragmented mitochondria after treating them with indicated concentration of MTZ, 8-OAc or Rote compared to the control DMSO. Statistical analysis was performed by one-way ANOVA using GraphPad Prism 10. ns = not significant, * = $p < 0.03$; ** = $p < 0.002$; *** = $p < 0.0002$; **** = $p < 0.0001$

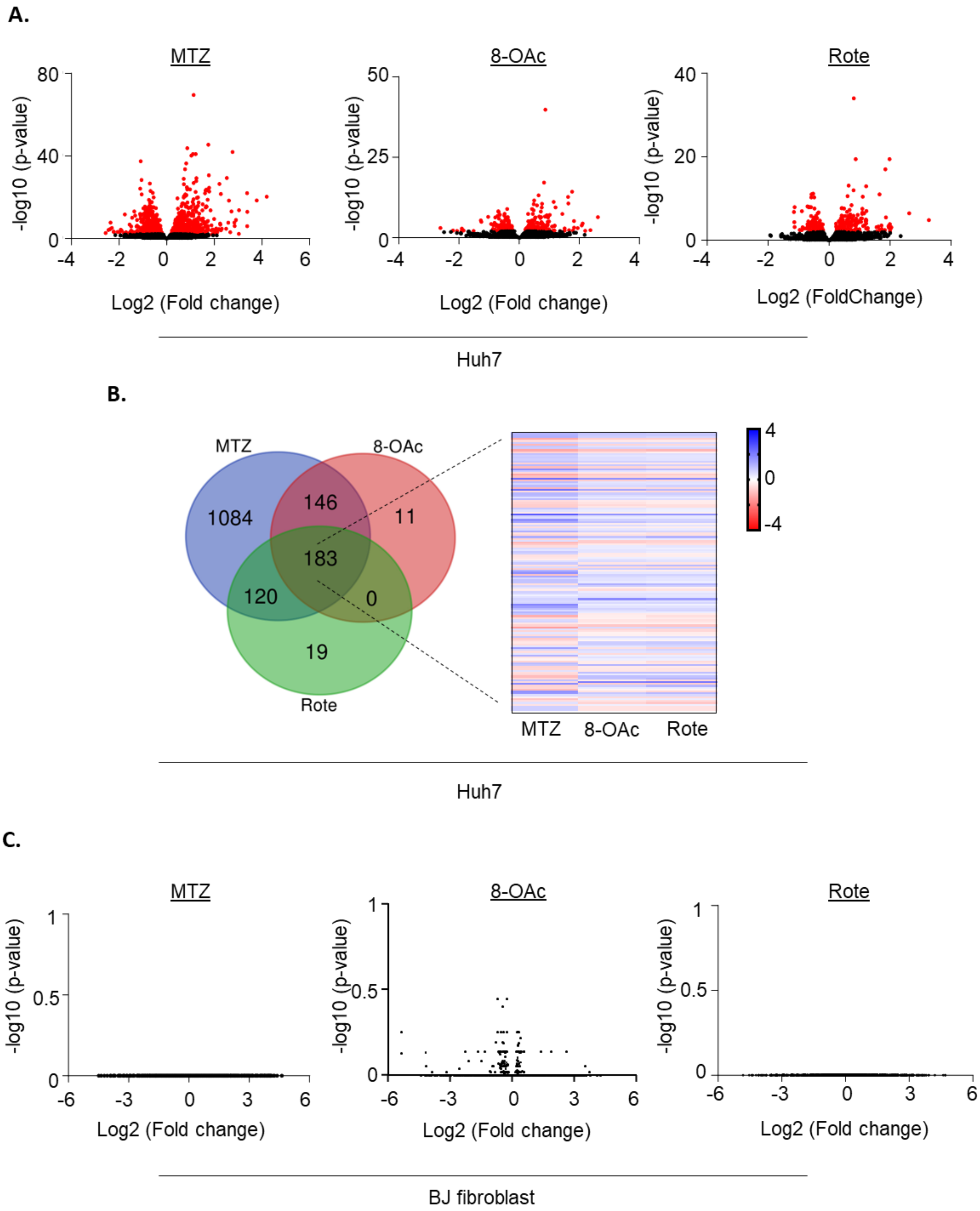
A.
MTZ
8-OAc
Rote
-log10 (p-value)
Log2 (Fold change)
Log2 (FoldChange)
Huh7
B.
MTZ
8-OAc
Rote
1084
146
11
183
120
0
19
4
0
-4
Huh7
C.
MTZ
8-OAc
Rote
-log10 (p-value)
Log2 (Fold change)
BJ fibroblast

Fig: S4

Fig. s4. MTZ, 8-OAc, and Rote alter gene expression in cancer cells, but not in non-cancer cells.

(A) Volcano plots representing changes in gene expression in Huh7 liver carcinoma cells treated with 10 μM MTZ, 8-OAc, and Rote for 24 hours. Red dots indicate significantly differentially expressed genes. Data was analyzed on 3 biological replicates per condition. (B) Venn diagram representing the overlap of significantly differentially expressed genes in indicated treatment conditions. (C) Heatmap showing the expression of overlapping differentially expressed genes shared between MTZ, 8-OAc, and Rote. (D) Volcano plots representing changes in gene expression in BJ fibroblasts treated with 10 μM MTZ, 8-OAc, and Rote for 24 hours.

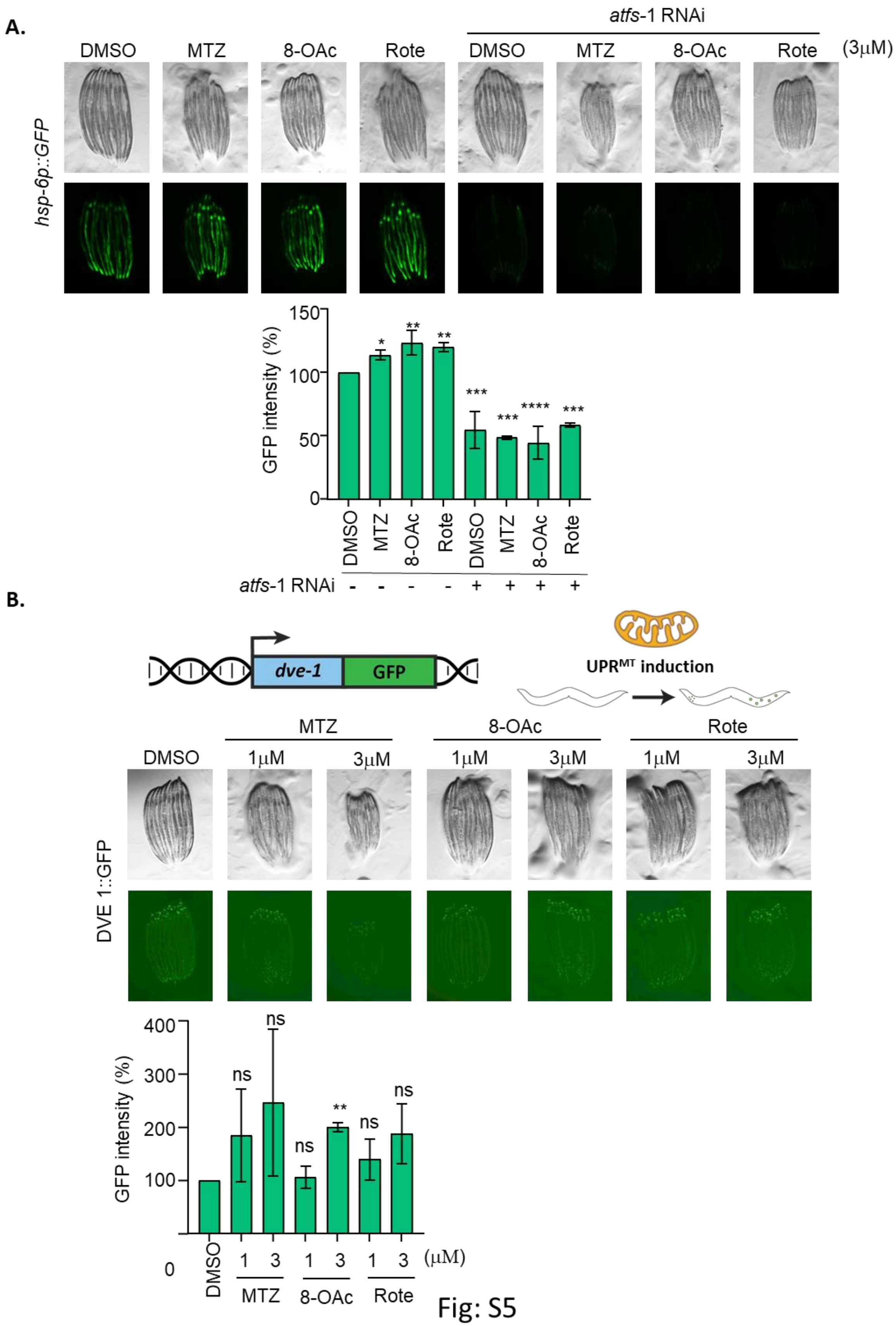
A.
atfs-1 RNAi
DMSO
MTZ
8-OAc
Rote
DMSO
MTZ
8-OAc
Rote
(3μM)
hsp-6p::GFP
150
100
50
0
GFP intensity (%)
*
**
**
***
***
****
***
DMSO
MTZ
8-OAc
Rote
DMSO
MTZ
8-OAc
Rote
atfs-1 RNAi - - - - + + + +
B.
dve-1
GFP
UPR^MT induction
MTZ
8-OAc
Rote
DMSO
1μM
3μM
1μM
3μM
1μM
3μM
DVE 1::GFP
400
300
200
100
0
GFP intensity (%)
ns
ns
ns
**
ns
ns
DMSO
1
3
1
3
1
3
(μM)
MTZ
8-OAc
Rote

Fig: S5

Fig. s5. Low concentrations of MTZ, 8-OAc, and Rote induce UPR^{MT} in an *atfs-1* dependent manner. (A) Representative fluorescent micrograph of UPR^{MT} transcriptional reporter worms (*hsp-6p::GFP*) of day 1 adult animals grown on empty vector or *atfs-1* RNAi plates supplemented with 3 µM MTZ, 8-OAc, or Rote for 24 hours from L1. The corresponding bar graph shows the graphical representation of change in GFP intensity of the worms treated with indicated concentration of MTZ, 8-OAc or Rote compared to the control DMSO. (B) Representative fluorescent micrographs of day 1 adult DVE-1::GFP worms grown on NGM plates supplemented with DMSO or indicated concentrations of MTZ, 8-OAc, or Rote for 24 hours from L1. The associated bar graph visually represents the change in GFP intensity observed in worms treated with specified concentrations of MTZ, 8-OAc, or Rote, compared to the control DMSO. All the statistical analysis was performed by one-way ANOVA using GraphPad Prism 10. ns = not significant, * = $p < 0.03$; ** = $p < 0.002$; *** = $p < 0.0002$; **** = $p < 0.0001$

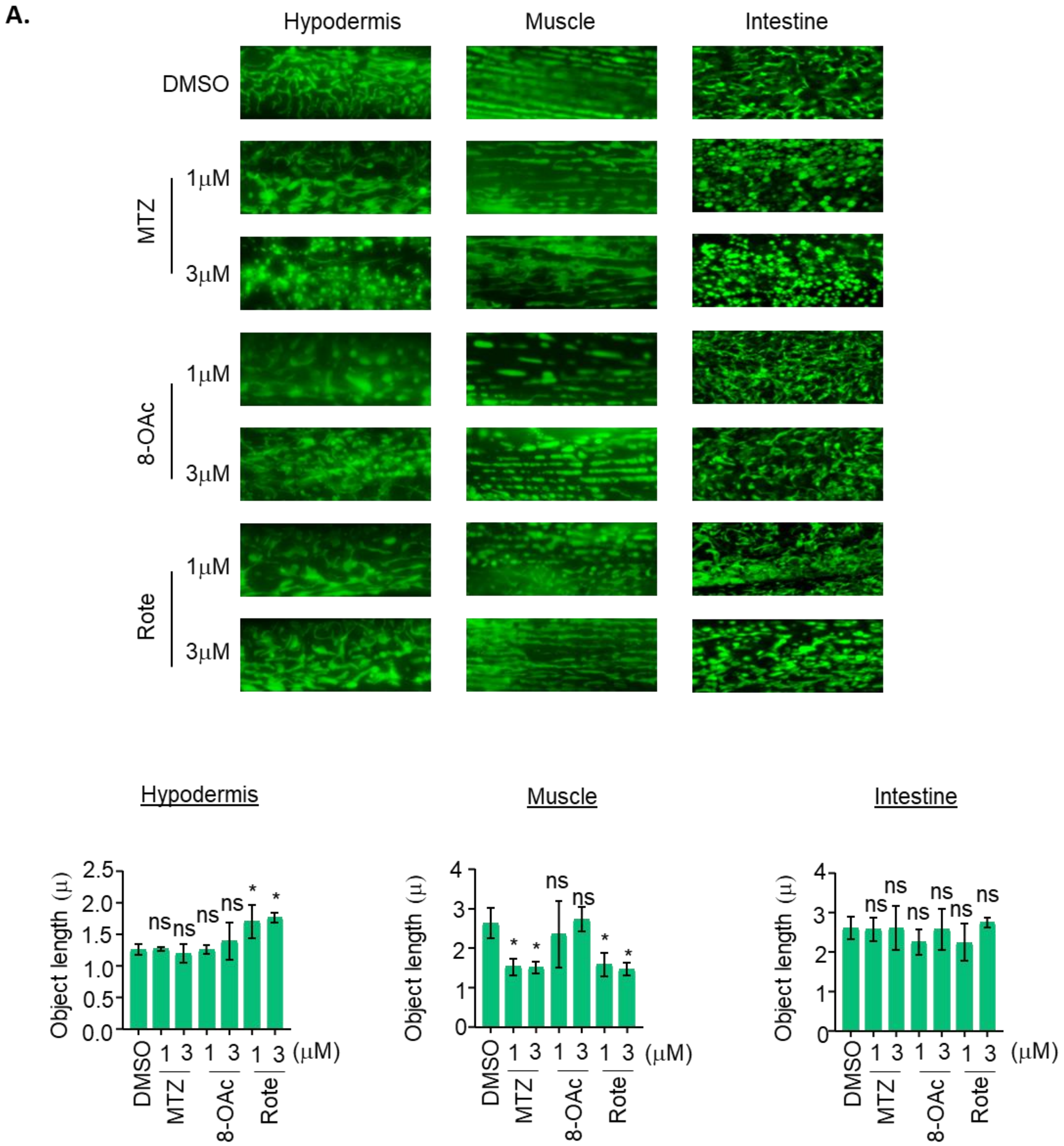

A.
Hypodermis
Muscle
Intestine
DMSO
MTZ
1μM
3μM
8-OAc
1μM
3μM
Rote
1μM
3μM
Hypodermis
Muscle
Intestine
Object length (μ)
DMSO
MTZ
8-OAc
Rote
1 3 1 3 1 3 (μM)
ns
*

Fig: S6

Fig. s6. Low concentrations of MTZ, 8-OAc, and Rote induce mitochondrial fragmentation. Representative fluorescence micrographs of MLS::GFP expressed in the hypodermis (*col-19p*), muscle (*myo-3p*), and intestine (*gly-19p*) of day 1 adult worms grown on NGM plates supplemented with DMSO or indicated concentrations of MTZ, 8-OAc, or Rote for 24 hours from L1. The mitochondrial length of each experimental conditions was measured by using the MitoMAPR imageJ macro and represented as a bar graph. Statistical analysis was performed by one-way ANOVA using GraphPad Prism 10. ns = not significant, * = $p < 0.03$; ** = $p < 0.002$; *** = $p < 0.0002$; **** = $p < 0.0001$

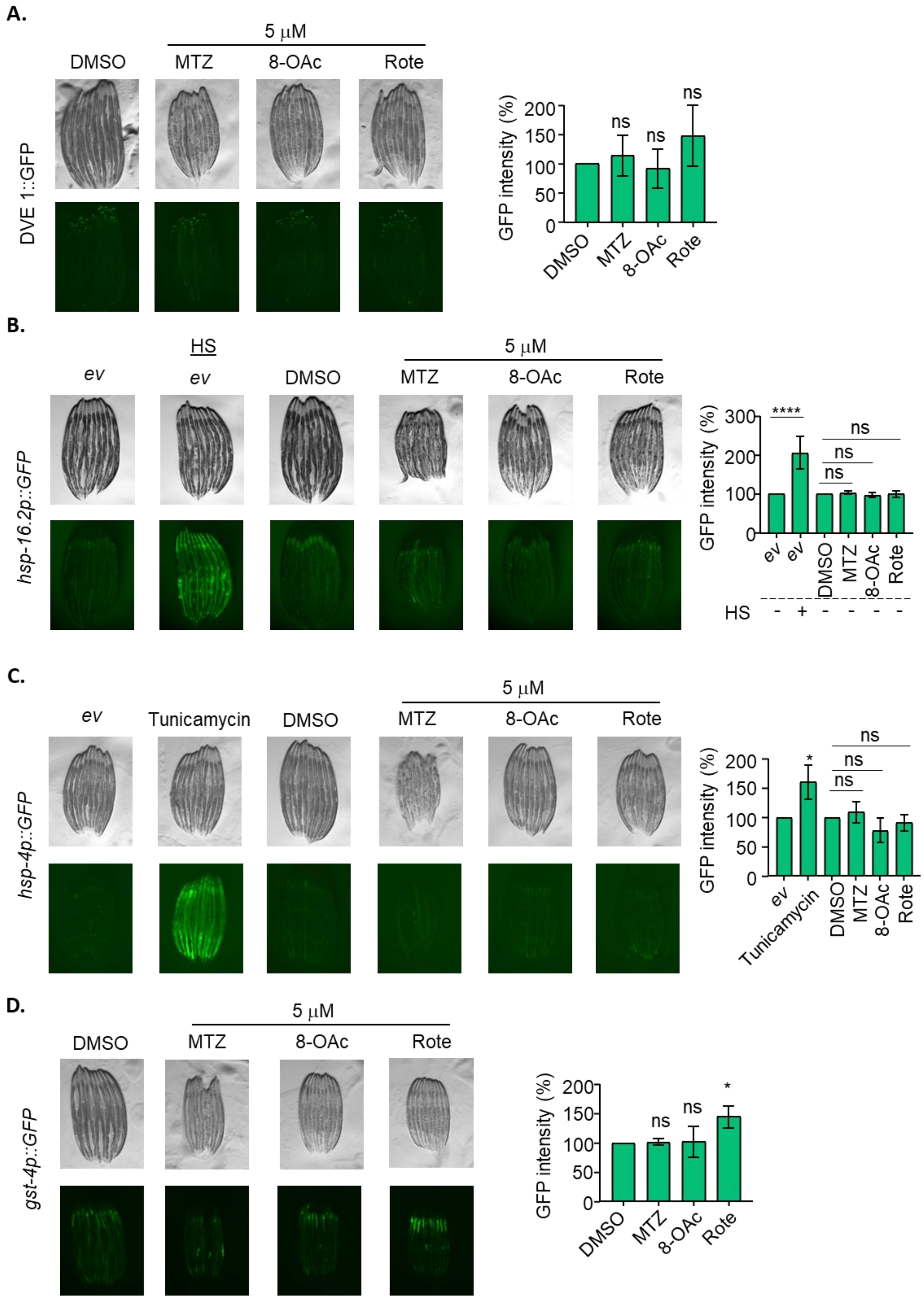
A.
5 μM
DMSO
MTZ
8-OAc
Rote
DVE 1::GFP
GFP intensity (%)
ns
B.
HS
ev
hsp-16.2p::GFP
****
HS
C.
Tunicamycin
hsp-4p::GFP
*
D.
gst-4p::GFP

Fig: S7

Fig. s7. High concentrations of MTZ, 8-OAc, and Rote do not induce reporters for stress responses.

(A) Representative fluorescent micrographs of DVE-1::GFP of day 2 adult animals grown on 5 µM MTZ, 8-OAc, or Rote for 24 hours from day 1 of adulthood. (B) Representative fluorescent micrograph of HSR transcriptional reporter worms (*hsp-16.2p::GFP*) of day 2 adult animals grown on 5 µM MTZ, 8-OAc, or Rote for 24 hours from day 1 of adulthood. Heat-shock for 2 hours at 34°C is used as a positive control. (C) Representative fluorescent micrograph of UPR^{ER} transcriptional reporter worms (*hsp-4p::GFP*) of day 2 adult animals grown on 5 µM MTZ, 8-OAc, or Rote for 24 hours from day 1 of adulthood. Growth on tunicamycin plates is used as a positive control. (D) Representative fluorescent micrograph of -transcriptional reporter worms for oxidative stress response (*gst-4p::GFP*) of day 2 adult animals grown on 5 µM MTZ, 8-OAc, or Rote for 24 hours from day 1 of adulthood. The GFP intensity of all experimental conditions of (A), (B), (C), and (D) were quantified and plotted as a bar graph. All statistical analyses were performed by one-way ANOVA using GraphPad Prism 10. ns = not significant, * = p < 0.03; ** = p <0.002; *** = p<0.0002; **** = p< 0.0001
