# Supplementary material for: Investigating impacts of the mycothiazole chemotype as a chemical probe for the study of mitochondrial function and aging
Source: GeroScience. 2024 Apr 3;46(6):6009–28. doi: 10.1007/s11357-024-01144-w (PMC11493899; doi:10.1007/s11357-024-01144-w)
Supplement: Supplementary file 2 — (DOCX 26 kb) [file 11357_2024_1144_MOESM2_ESM.docx]

**Tab. s1: GO biological processes involve upon MTZ, 8-Oac, Rote in Huh7 cells**

| **Mycothiazole** | |  |  |  |  |
| --- | --- | --- | --- | --- | --- |
| **Index** | **Name** | **P-value** | **Adjusted p-value** | **Odds Ratio** | **Combined score** |
| 1 | Cytoplasmic Translation (GO:0002181) | 1.23E-34 | 4.06E-31 | 18.96 | 1480.71 |
| 2 | Macromolecule Biosynthetic Process (GO:0009059) | 3.40E-23 | 5.63E-20 | 7.28 | 376.67 |
| 3 | Peptide Biosynthetic Process (GO:0043043) | 8.31E-22 | 9.17E-19 | 7.68 | 372.84 |
| 4 | Gene Expression (GO:0010467) | 4.53E-21 | 3.75E-18 | 5.06 | 236.89 |
| 5 | Translation (GO:0006412) | 1.16E-19 | 7.70E-17 | 5.53 | 241.12 |
| 6 | protein-RNA Complex Assembly (GO:0022618) | 1.39E-13 | 7.67E-11 | 5.59 | 165.48 |
| 7 | Regulation Of Translation (GO:0006417) | 2.55E-12 | 1.21E-09 | 4.43 | 118.14 |
| 8 | mRNA Splicing, Via Spliceosome (GO:0000398) | 4.27E-11 | 1.77E-08 | 4.06 | 96.82 |
| 9 | RNA Splicing, Via Transesterification Reactions With Bulged Adenosine As Nucleophile (GO:0000377) | 1.43E-10 | 5.26E-08 | 4.27 | 96.79 |
| 10 | mRNA Processing (GO:0006397) | 2.62E-10 | 8.66E-08 | 3.85 | 85 |
| 11 | DNA-templated DNA Replication (GO:0006261) | 1.01E-09 | 2.96E-07 | 7.16 | 148.39 |
| 12 | Ribonucleoprotein Complex Biogenesis (GO:0022613) | 1.07E-09 | 2.96E-07 | 5.09 | 105.05 |
| 13 | RNA Processing (GO:0006396) | 3.82E-09 | 9.72E-07 | 3.87 | 75.02 |
| 14 | DNA Metabolic Process (GO:0006259) | 9.00E-09 | 0.000002129 | 3.07 | 56.93 |
| 15 | Ribosome Biogenesis (GO:0042254) | 2.08E-08 | 0.000004595 | 3.99 | 70.61 |
| 16 | Regulation Of Apoptotic Process (GO:0042981) | 3.70E-08 | 0.000007648 | 2.16 | 36.93 |
| 17 | Mitotic DNA Replication (GO:1902969) | 9.41E-08 | 0.00001755 | 43.58 | 705.03 |
| 18 | RNA Splicing (GO:0008380) | 9.55E-08 | 0.00001755 | 4.83 | 78.1 |
| 19 | Formation Of Cytoplasmic Translation Initiation Complex (GO:0001732) | 1.04E-07 | 0.00001811 | 24.92 | 400.71 |
| 20 | DNA Replication (GO:0006260) | 1.15E-07 | 0.00001909 | 5.71 | 91.21 |
| 21 | Cytoplasmic Translational Initiation (GO:0002183) | 1.32E-07 | 0.00002085 | 10.85 | 171.78 |
| 22 | Mitotic Sister Chromatid Segregation (GO:0000070) | 1.80E-07 | 0.00002708 | 4.4 | 68.31 |
| 23 | Ribosomal Small Subunit Biogenesis (GO:0042274) | 1.91E-07 | 0.00002748 | 5.13 | 79.4 |
| 24 | Carboxylic Acid Transport (GO:0046942) | 2.84E-07 | 0.00003918 | 6.74 | 101.56 |
| 25 | DNA Replication Initiation (GO:0006270) | 3.89E-07 | 0.00005155 | 14.03 | 207.03 |

| **8-OAc** |  |  |  |  |  |
| --- | --- | --- | --- | --- | --- |
| **Index** | **Name** | **P-value** | **Adjusted p-value** | **Odds Ratio** | **Combined score** |
| 1 | Cytoplasmic Translation (GO:0002181) | 7.80E-16 | 1.15E-12 | 20.02 | 696.32 |
| 2 | Macromolecule Biosynthetic Process (GO:0009059) | 6.67E-11 | 4.91E-08 | 9.12 | 213.74 |
| 3 | Translation (GO:0006412) | 4.07E-10 | 2.00E-07 | 7.44 | 160.82 |
| 4 | Neutral Amino Acid Transport (GO:0015804) | 6.17E-09 | 0.000001826 | 25.54 | 482.72 |
| 5 | Peptide Biosynthetic Process (GO:0043043) | 6.20E-09 | 0.000001826 | 8.55 | 161.64 |
| 6 | Carbohydrate Catabolic Process (GO:0016052) | 2.36E-08 | 0.00000578 | 20.89 | 366.85 |
| 7 | Amino Acid Transport (GO:0006865) | 6.11E-08 | 0.00001286 | 18.13 | 301.2 |
| 8 | Regulation Of Translation (GO:0006417) | 1.39E-07 | 0.00002489 | 6.54 | 103.19 |
| 9 | Hexose Biosynthetic Process (GO:0019319) | 1.52E-07 | 0.00002489 | 21.45 | 336.74 |
| 10 | Carboxylic Acid Transport (GO:0046942) | 1.93E-07 | 0.00002847 | 15.31 | 236.63 |
| 11 | Glycolytic Process (GO:0006096) | 9.83E-07 | 0.0001315 | 22.29 | 308.37 |
| 12 | Gluconeogenesis (GO:0006094) | 0.00000182 | 0.0002201 | 19.72 | 260.6 |
| 13 | Pyruvate Metabolic Process (GO:0006090) | 1.944E-06 | 0.0002201 | 13.96 | 183.55 |
| 14 | Gene Expression (GO:0010467) | 2.693E-06 | 0.0002831 | 4.68 | 60.08 |
| 15 | Positive Regulation Of Translation (GO:0045727) | 5.592E-06 | 0.0005488 | 7.84 | 94.82 |
| 16 | Glucose Metabolic Process (GO:0006006) | 6.065E-06 | 0.0005579 | 11.54 | 138.59 |
| 17 | Alanine Transport (GO:0032328) | 8.831E-06 | 0.0007647 | 42.39 | 493.33 |
| 18 | Amino Acid Transmembrane Transport (GO:0003333) | 9.541E-06 | 0.0007802 | 14.23 | 164.53 |
| 19 | Positive Regulation Of Chromosome Separation (GO:1905820) | 0.00001264 | 0.0009791 | 37.68 | 424.99 |
| 20 | Cytoplasmic Translational Initiation (GO:0002183) | 0.00002506 | 0.001844 | 17.02 | 180.27 |
| 21 | Regulation Of Mitotic Cytokinesis (GO:1902412) | 0.00003201 | 0.002243 | 84.44 | 873.96 |
| 22 | Regulation Of Cellular Respiration (GO:0043457) | 0.00004053 | 0.002486 | 26.08 | 263.77 |
| 23 | Regulation Of Oxidative Phosphorylation (GO:0002082) | 0.00004053 | 0.002486 | 26.08 | 263.77 |
| 24 | Regulation Of Aerobic Respiration (GO:1903715) | 0.00004053 | 0.002486 | 26.08 | 263.77 |
| 25 | Sulfur Amino Acid Transport (GO:0000101) | 0.00005552 | 0.003269 | 63.33 | 620.56 |
|  |  |  |  |  |  |
|  |  |  |  |  |  |
|  |  |  |  |  |  |

| **Rotenone** | |  |  |  |  |
| --- | --- | --- | --- | --- | --- |
| **Index** | **Name** | **P-value** | **Adjusted p-value** | **Odds Ratio** | **Combined score** |
| 1 | Cytoplasmic Translation (GO:0002181) | 2.35E-12 | 3.51E-09 | 16.54 | 442.98 |
| 2 | Macromolecule Biosynthetic Process (GO:0009059) | 2.54E-09 | 0.000001896 | 8.34 | 165 |
| 3 | Translation (GO:0006412) | 1.01E-08 | 0.000005049 | 6.87 | 126.43 |
| 4 | Peptide Biosynthetic Process (GO:0043043) | 2.87E-08 | 0.00001074 | 8.3 | 144.16 |
| 5 | Carbohydrate Catabolic Process (GO:0016052) | 3.37E-07 | 0.0001009 | 18.64 | 277.83 |
| 6 | Mitotic Chromosome Condensation (GO:0007076) | 4.72E-07 | 0.0001086 | 44.92 | 654.31 |
| 7 | Mitotic Sister Chromatid Segregation (GO:0000070) | 5.09E-07 | 0.0001086 | 9.06 | 131.29 |
| 8 | Regulation Of Chromosome Segregation (GO:0051983) | 5.81E-07 | 0.0001086 | 24.6 | 353.21 |
| 9 | Positive Regulation Of Cell Cycle Process (GO:0090068) | 8.98E-07 | 0.0001493 | 8.47 | 117.93 |
| 10 | Chromosome Condensation (GO:0030261) | 1.346E-06 | 0.0001921 | 20.81 | 281.33 |
| 11 | Gene Expression (GO:0010467) | 1.412E-06 | 0.0001921 | 4.96 | 66.75 |
| 12 | Hexose Biosynthetic Process (GO:0019319) | 2.343E-06 | 0.0002921 | 18.65 | 241.84 |
| 13 | Cell Cycle G2/M Phase Transition (GO:0044839) | 5.287E-06 | 0.0006085 | 15.91 | 193.28 |
| 14 | Regulation Of Chromosome Separation (GO:1905818) | 7.192E-06 | 0.0007685 | 44.72 | 529.62 |
| 15 | Positive Regulation Of Chromosome Separation (GO:1905820) | 0.0000103 | 0.001027 | 39.75 | 456.48 |
| 16 | Glycolytic Process (GO:0006096) | 0.00001642 | 0.001467 | 18.7 | 206.06 |
| 17 | Regulation Of Cell Cycle Process (GO:0010564) | 0.00001667 | 0.001467 | 6.77 | 74.46 |
| 18 | Negative Regulation Of Blood Coagulation (GO:0030195) | 0.00001953 | 0.001569 | 17.95 | 194.69 |
| 19 | Pyruvate Metabolic Process (GO:0006090) | 0.00001993 | 0.001569 | 12.29 | 132.97 |
| 20 | Gluconeogenesis (GO:0006094) | 0.00002709 | 0.001994 | 16.62 | 174.81 |
| 21 | Carboxylic Acid Transport (GO:0046942) | 0.000028 | 0.001994 | 11.5 | 120.56 |
| 22 | Positive Regulation Of Chromosome Organization (GO:2001252) | 0.00003164 | 0.002138 | 16.03 | 166.07 |
| 23 | Regulation Of Cytokinesis (GO:0032465) | 0.00003288 | 0.002138 | 8.67 | 89.46 |
| 24 | Positive Regulation Of Chromosome Segregation (GO:0051984) | 0.00004216 | 0.002445 | 25.55 | 257.37 |
| 25 | Intracellular Lipid Transport (GO:0032365) | 0.00004216 | 0.002445 | 25.55 | 257.37 |
|  |  |  |  |  |  |
