# Supplementary material for: Investigating impacts of the mycothiazole chemotype as a chemical probe for the study of mitochondrial function and aging
Source: GeroScience. 2024 Apr 3;46(6):6009–28. doi: 10.1007/s11357-024-01144-w (PMC11493899; doi:10.1007/s11357-024-01144-w)
Supplement: Supplementary file 3 — (DOCX 25 kb) [file 11357_2024_1144_MOESM3_ESM.docx]

**Tab. s2: Biological processes involved in MTZ, 8-Oac, Rote treatment in worms.**

| **Mycothiazole** | |  |  |  |  |
| --- | --- | --- | --- | --- | --- |
| Index | Name | P-value | Adjusted p-value | Z-score | Combined score |
| 1 | Defense response to bacterium (GO:0042742) | 1.11E-11 | 5.76E-09 | -1.47 | 37.19 |
| 2 | energy coupled proton transmembrane transport, against electrochemical gradient (GO:0015988) | 6.06E-05 | 0.002609 | -3.13 | 30.37 |
| 3 | ATP hydrolysis coupled proton transport (GO:0015991) | 0.000571 | 0.01019 | -3.81 | 28.44 |
| 4 | cellular sodium ion homeostasis (GO:0006883) | 0.000106 | 0.003435 | -2.92 | 26.71 |
| 5 | Respiratory electron transport chain (GO:0022904) | 7.88E-06 | 0.000814 | -2.26 | 26.57 |
| 6 | sodium ion export from cell (GO:0036376) | 0.000106 | 0.003435 | -2.88 | 26.35 |
| 7 | ATP hydrolysis coupled cation transmembrane transport (GO:0099132) | 0.000571 | 0.01019 | -3.45 | 25.75 |
| 8 | defense response to Gram-positive bacterium (GO:0050830) | 4.11E-08 | 7.09E-06 | -1.49 | 25.39 |
| 9 | negative regulation of lipid storage (GO:0010888) | 0.000571 | 0.01019 | -3.39 | 25.33 |
| 10 | sodium ion export (GO:0071436) | 0.000106 | 0.003435 | -2.76 | 25.24 |
| 11 | cellular monovalent inorganic cation homeostasis (GO:0030004) | 0.000173 | 0.004702 | -2.91 | 25.17 |
| 12 | mitochondrial transport (GO:0006839) | 1.58E-07 | 2.04E-05 | -1.61 | 25.15 |
| 13 | cellular potassium ion homeostasis (GO:0030007) | 0.000106 | 0.003435 | -2.64 | 24.15 |
| 14 | tryptophan catabolic process (GO:0006569) | 0.000571 | 0.01019 | -3.23 | 24.15 |
| 15 | negative regulation of lipid localization (GO:1905953) | 0.000571 | 0.01019 | -3.19 | 23.81 |
| 16 | muscle contraction (GO:0006936) | 5.93E-05 | 0.002609 | -2.35 | 22.85 |
| 17 | sodium ion homeostasis (GO:0055078) | 0.000265 | 0.006223 | -2.76 | 22.74 |
| 18 | acyl-CoA metabolic process (GO:0006637) | 0.000135 | 0.003872 | -2.23 | 19.86 |
| 19 | carbohydrate transport (GO:0008643) | 0.000265 | 0.006223 | -2.35 | 19.32 |
| 20 | methionine metabolic process (GO:0006555) | 0.000977 | 0.01601 | -2.75 | 19.08 |
| 21 | monocarboxylic acid metabolic process (GO:0032787) | 4.50E-05 | 0.002586 | -1.9 | 18.97 |
| 22 | cellular protein catabolic process (GO:0044257) | 1.55E-05 | 0.001337 | -1.63 | 18.06 |
| 23 | defense response to Gram-negative bacterium (GO:0050829) | 3.42E-08 | 7.09E-06 | -1.05 | 17.99 |
| 24 | potassium ion homeostasis (GO:0055075) | 0.001289 | 0.01754 | -2.58 | 17.2 |
| 25 | mitochondrial ATP synthesis coupled electron transport (GO:0042775) | 3.93E-05 | 0.002538 | -1.68 | 17.09 |

| **8-Oac** |  |  |  |  |  |
| --- | --- | --- | --- | --- | --- |
| Index | Name | P-value | Adjusted p-value | Z-score | Combined score |
| 1 | medium-chain fatty acid metabolic process (GO:0051791) | 0.001129 | 0.02273 | -3.89 | 26.39 |
| 2 | medium-chain fatty acid catabolic process (GO:0051793) | 0.001129 | 0.02273 | -3.78 | 25.63 |
| 3 | carbohydrate transport (GO:0008643) | 0.000105 | 0.004933 | -2.38 | 21.78 |
| 4 | defense response to bacterium (GO:0042742) | 4.61E-07 | 6.51E-05 | -1.47 | 21.51 |
| 5 | defense response to Gram-positive bacterium (GO:0050830) | 1.59E-05 | 0.001118 | -1.5 | 16.54 |
| 6 | cysteine biosynthetic process (GO:0019344) | 0.003308 | 0.05182 | -2.67 | 15.26 |
| 7 | heat acclimation (GO:0010286) | 0.00402 | 0.05668 | -2.68 | 14.76 |
| 8 | response to reactive oxygen species (GO:0000302) | 0.001042 | 0.02273 | -2.1 | 14.4 |
| 9 | monocarboxylic acid metabolic process (GO:0032787) | 0.000912 | 0.02273 | -1.92 | 13.41 |
| 10 | hydrogen peroxide metabolic process (GO:0042743) | 0.05166 | 0.1817 | -4.23 | 12.55 |
| 11 | hydrogen peroxide catabolic process (GO:0042744) | 0.05166 | 0.1817 | -3.87 | 11.46 |
| 12 | response to hydrogen peroxide (GO:0042542) | 0.007499 | 0.09612 | -2.27 | 11.12 |
| 13 | pyruvate metabolic process (GO:0006090) | 0.01074 | 0.1082 | -2.37 | 10.73 |
| 14 | ammonium transmembrane transport (GO:0072488) | 0.06001 | 0.1817 | -3.79 | 10.65 |
| 15 | cortical actin cytoskeleton organization (GO:0030866) | 0.008521 | 0.1001 | -2.2 | 10.49 |
| 16 | water transport (GO:0006833) | 0.05166 | 0.1817 | -3.53 | 10.45 |
| 17 | phenol-containing compound biosynthetic process (GO:0046189) | 0.05166 | 0.1817 | -3.3 | 9.79 |
| 18 | response to UV-C (GO:0010225) | 0.05166 | 0.1817 | -3.29 | 9.74 |
| 19 | alpha-amino acid catabolic process (GO:1901606) | 0.002662 | 0.04691 | -1.62 | 9.62 |
| 20 | fluid transport (GO:0042044) | 0.05166 | 0.1817 | -3.24 | 9.59 |
| 21 | tryptophan catabolic process (GO:0006569) | 0.05166 | 0.1817 | -3.22 | 9.55 |
| 22 | muscle contraction (GO:0006936) | 0.01726 | 0.1431 | -2.31 | 9.36 |
| 23 | long-chain fatty acid metabolic process (GO:0001676) | 0.05166 | 0.1817 | -3.14 | 9.29 |
| 24 | hexose biosynthetic process (GO:0019319) | 0.06828 | 0.1817 | -3.44 | 9.23 |
| 25 | glucan catabolic process (GO:0009251) | 0.05166 | 0.1817 | -2.98 | 8.8 |

| **Rotenone** | |  |  |  |  |
| --- | --- | --- | --- | --- | --- |
| Index | Name | P-value | Adjusted p-value | Z-score | Combined score |
| 1 | tryptophan catabolic process (GO:0006569) | 8.05E-05 | 0.004154 | -3.3 | 31.13 |
| 2 | carbohydrate transport (GO:0008643) | 2.02E-05 | 0.0013 | -2.38 | 25.68 |
| 3 | defense response to bacterium (GO:0042742) | 6.48E-08 | 1.67E-05 | -1.47 | 24.41 |
| 4 | medium-chain fatty acid metabolic process (GO:0051791) | 0.003736 | 0.05074 | -3.86 | 21.57 |
| 5 | medium-chain fatty acid catabolic process (GO:0051793) | 0.003736 | 0.05074 | -3.7 | 20.67 |
| 6 | mitochondrial transport (GO:0006839) | 5.88E-06 | 0.000511 | -1.61 | 19.43 |
| 7 | phenol-containing compound biosynthetic process (GO:0046189) | 0.003736 | 0.05074 | -3.33 | 18.62 |
| 8 | fatty acid beta-oxidation using acyl-CoA oxidase (GO:0033540) | 0.006827 | 0.05871 | -3.7 | 18.45 |
| 9 | defense response to Gram-positive bacterium (GO:0050830) | 5.94E-06 | 0.000511 | -1.49 | 17.97 |
| 10 | glucan catabolic process (GO:0009251) | 0.003736 | 0.05074 | -3 | 16.78 |
| 11 | response to gamma radiation (GO:0010332) | 0.001331 | 0.02862 | -2.45 | 16.25 |
| 12 | translational elongation (GO:0006414) | 0.001999 | 0.03684 | -2.51 | 15.58 |
| 13 | glycogen catabolic process (GO:0005980) | 0.005175 | 0.05563 | -2.92 | 15.39 |
| 14 | monocarboxylic acid metabolic process (GO:0032787) | 0.000388 | 0.01252 | -1.9 | 14.92 |
| 15 | methionine metabolic process (GO:0006555) | 0.005175 | 0.05563 | -2.79 | 14.66 |
| 16 | regulation of phosphorylation (GO:0042325) | 0.003736 | 0.05074 | -2.62 | 14.66 |
| 17 | establishment of protein localization to mitochondrion (GO:0072655) | 0.000135 | 0.005787 | -1.64 | 14.61 |
| 18 | protein import into mitochondrial matrix (GO:0030150) | 0.001999 | 0.03684 | -2.27 | 14.13 |
| 19 | tyrosine metabolic process (GO:0006570) | 0.006827 | 0.05871 | -2.77 | 13.79 |
| 20 | aromatic amino acid family catabolic process (GO:0009074) | 0.008685 | 0.06056 | -2.88 | 13.67 |
| 21 | cellular sodium ion homeostasis (GO:0006883) | 0.008685 | 0.06056 | -2.83 | 13.45 |
| 22 | aspartate family amino acid catabolic process (GO:0009068) | 0.005175 | 0.05563 | -2.55 | 13.4 |
| 23 | sodium ion export from cell (GO:0036376) | 0.008685 | 0.06056 | -2.81 | 13.36 |
| 24 | regulation of mitochondrial membrane permeability (GO:0046902) | 0.006827 | 0.05871 | -2.67 | 13.31 |
| 25 | cell part morphogenesis (GO:0032990) | 0.006827 | 0.05871 | -2.63 | 13.1 |
