# Supplementary material for: Investigating impacts of the mycothiazole chemotype as a chemical probe for the study of mitochondrial function and aging
Source: GeroScience. 2024 Apr 3;46(6):6009–28. doi: 10.1007/s11357-024-01144-w (PMC11493899; doi:10.1007/s11357-024-01144-w)
Supplement: Supplementary file 4 — (DOCX 13 kb) [file 11357_2024_1144_MOESM4_ESM.docx]

**Tab. s3: *C elegans* strains used in this study.**

| **Strains Used in this study** | **Source** | **Notes** |
| --- | --- | --- |
|  |  |  |
| *C elegans*: Bristol (N2) strains wild type (WT) | CGC | WT |
| *C. elegans*: CL2166: dvIs19[pAG15(gst-4p::GFP::NLS)] III | CGC |  |
| *C. elegans:* CL2070: *dvIs70[pCL25 (hsp-16.2p::GFP), pRF4(rol-6)]* | CGC |  |
| *C elegans*: AGD1988: zcls13[hsp-6p::GFP] | Garcia et.al. 2022 |  |
| *C elegans*: RHS19: glp-4(bn2)I | This study | 6X |
| *C elegans*: SJ4197: zcls39[dve-1p::dve-1::GFP]II | Dillin lab | 3X |
| *C elegans*: AGD2053: zcIs4[hsp-4P::GFP]V | Dillin lab | 9X |
| *C elegans*: AGD1664: uthSi17[myo-3p::MLS::GFP::unc-54 3’UTR::cb-unc-119(+)] I; unc-119(ed3) III | Dillin lab | N/A |
| *C elegans*: AGD2837: uthSi83[col-19p::MLS::GFP(65C)::unc-54 3'UTR, cb-unc-119(+)] I; unc-119(ed3) III; | Dillin lab | N/A |
| *C elegans*: AGD2838: uthSi84[vha-6p::MLS::GFP(65C)::unc-54 3'UTR, cb-unc-119(+)] I; unc-119(ed3) III; | Dillin lab | N/A |
