# Supplementary material for: Investigating impacts of the mycothiazole chemotype as a chemical probe for the study of mitochondrial function and aging
Source: GeroScience. 2024 Apr 3;46(6):6009–28. doi: 10.1007/s11357-024-01144-w (PMC11493899; doi:10.1007/s11357-024-01144-w)
Supplement: Supplementary file 5 — (DOCX 25 kb) [file 11357_2024_1144_MOESM5_ESM.docx]

| **Supplementary Table 4: Statistical analysis of lifespan** | |  |  |  |  |
| --- | --- | --- | --- | --- | --- |
|  |  |  |  |  |  |
| **Corresponding Figure** | **Strain, Treatment** | **Median Lifespan [days]** | **#Death/ #Total** | **% change in median lifespan** | **P-value vs N2 Log-rank (Mantel-Cox)** |
|  |  |  |  |  |  |
| Fig 2E | N2, DMSO | 20 | 118/120 | -- | -- |
|  | N2, 1mM MTZ | 22 | 119/120 | 10 | 0.0004 |
|  | N2, 3mM MTZ | 20 | 117/120 | -- | 0.0007 |
|  | N2, 1mM 8-Oac | 20 | 115/120 | -- | 0.7338 |
|  | N2, 3mM 8-Oac | 22 | 119/120 | 10 | <0.0001 |
|  | N2, 1mM Rote | 20 | 119/120 | -- | 0.2991 |
|  | N2, 3mM Rote | 22 | 111/120 | 10 | <0.0001 |
|  |  |  |  |  |  |
| Fig 2E | N2, DMSO | 20 | 115/120 | -- | -- |
|  | N2, 1mM MTZ | 22 | 117/120 | 10 | <0.0001 |
|  | N2, 3mM MTZ | 22 | 105/120 | 10 | <0.0001 |
|  | N2, 1mM 8-Oac | 20 | 105/120 | -- | 0.4241 |
|  | N2, 3mM 8-Oac | 20 | 109/120 | -- | 0.0133 |
|  | N2, 1mM Rote | 22 | 116/120 | 10 | 0.0001 |
|  | N2, 3mM Rote | 20 | 120/120 | -- | 0.5967 |
|  |  |  |  |  |  |
| Fig 2E | N2, DMSO | 18 | 118/120 | -- | -- |
|  | N2, 1mM MTZ | N/A | N/A | N/A | N/A |
|  | N2, 3mM MTZ | N/A | N/A | N/A | N/A |
|  | N2, 1mM 8-Oac | 20 | 113/120 | 11.11111111 | <0.0001 |
|  | N2, 3mM 8-Oac | 20 | 114/120 | 11.11111111 | <0.0001 |
|  | N2, 1mM Rote | 22 | 113/120 | 22.22222222 | <0.0001 |
|  | N2, 3mM Rote | 20 | 116/120 | 11.11111111 | <0.0001 |
|  |  |  |  |  |  |
| Fig 3C | N2, DMSO | 14 | 88/120 | -- | -- |
|  | N2, 5mM MTZ | 15 | 87/120 | 7.142857143 | 0.0001 |
|  | N2, 5mM 8-Oac | 21 | 76/120 | 50 | <0.0001 |
|  | N2, 5mM Rote | 25 | 86/120 | 78.57142857 | <0.0001 |
|  |  |  |  |  |  |
| Fig 3C | N2, DMSO | 17 | 97/100 | -- | -- |
|  | N2, 5mM MTZ | 21 | 97/100 | 23.52941176 | <0.0001 |
|  | N2, 5mM 8-Oac | 20 | 98/100 | 17.64705882 | 0.0268 |
|  | N2, 5mM Rote | 24 | 94/100 | 41.17647059 | <0.0001 |
|  |  |  |  |  |  |
| Fig 5A | N2, DMSO, *atfs*-1 RNAi | 18 | 98/120 | -- | -- |
|  | N2, 5mM MTZ, *atfs-*1 RNAi | 4 | 100/120 | -77.77777778 | <0.0001 |
|  | N2, 5mM 8-Oac, *atfs-*1 RNAi | 22 | 104/120 | 22.22222222 | <0.0001 |
|  | N2, 5mM Rote, *atfs-*1 RNAi | 10 | 100/120 | -44.44444444 | <0.0001 |
|  |  |  |  |  |  |
| Fig 5A | N2, DMSO, *atfs*-1 RNAi | 15 | 98/100 | -- | -- |
|  | N2, 5mM MTZ, *atfs-*1 RNAi | 6 | 100/100 | -60 | <0.0001 |
|  | N2, 5mM 8-Oac, *atfs-*1 RNAi | 19 | 94/100 | 26.66666667 | 0.0002 |
|  | N2, 5mM Rote, *atfs-*1 RNAi | 13 | 99/100 | -13.33333333 | 0.5256 |
|  |  |  |  |  |  |
| Fig 5B | N2, DMSO, *hsf*-1 RNAi | 12 | 109/120 | -- | -- |
|  | N2, 5mM MTZ, *hsf-*1 RNAi | 5 | 115/120 | -58.33333333 | <0.0001 |
|  | N2, 5mM 8-Oac, *hsf-*1 RNAi | 10 | 112/120 | -16.66666667 | 0.0006 |
|  | N2, 5mM Rote, *hsf-*1 RNAi | 10 | 115/120 | -16.66666667 | 0.0017 |
|  |  |  |  |  |  |
| Fig 5B | N2, DMSO, *hsf*-1 RNAi | 12 | 78/100 | -- | -- |
|  | N2, 5mM MTZ, *hsf-*1 RNAi | 5 | 97/100 | -58.33333333 | <0.0001 |
|  | N2, 5mM 8-Oac, *hsf-*1 RNAi | 9 | 98/100 | -25 | <0.0001 |
|  | N2, 5mM Rote, *hsf-*1 RNAi | 9 | 100/100 | -25 | <0.0001 |
|  |  |  |  |  |  |
| Fig 5B | N2, DMSO, *hsf*-1 RNAi | 15 | 99/101 | -- | -- |
|  | N2, 5mM MTZ, *hsf-*1 RNAi | N/A | N/A | N/A | N/A |
|  | N2, 5mM 8-Oac, *hsf-*1 RNAi | 13 | 92/95 | -13.33333333 | 0.2007 |
|  | N2, 5mM Rote, *hsf-*1 RNAi | N/A | N/A | N/A | N/A |
|  |  |  |  |  |  |
| Fig 5C | N2, DMSO, *daf*-16 RNAi | 12 | 95/100 | -- | -- |
|  | N2, 5mM MTZ, *daf-*16 RNAi | 16 | 98/100 | 33.33333333 | <0.0001 |
|  | N2, 5mM 8-Oac, *daf-16* RNAi | 18 | 96/100 | 50 | <0.0001 |
|  | N2, 5mM Rote, *daf-16* RNAi | 16 | 97/100 | 33.33333333 | <0.0001 |
|  |  |  |  |  |  |
| Fig 5C | N2, DMSO, *daf*-16 RNAi | 12 | 99/100 | -- | -- |
|  | N2, 5mM MTZ, *daf-*16 RNAi | N/A | N/A | N/A | N/A |
|  | N2, 5mM 8-Oac, *daf-16* RNAi | 14 | 92/100 | 14.28571429 | <0.0001 |
|  | N2, 5mM Rote, *daf-16* RNAi | 13 | 91/100 | 8.333333333 | <0.0001 |
|  |  |  |  |  |  |
| Fig 5C | N2, DMSO, *daf*-16 RNAi | 12 | 100/100 | -- | -- |
|  | N2, 5mM MTZ, *daf-*16 RNAi | 14 | 97/100 | 14.28571429 | <0.0001 |
|  | N2, 5mM 8-Oac, *daf-16* RNAi | 14 | 99/100 | 14.28571429 | <0.0001 |
|  | N2, 5mM Rote, *daf-16* RNAi | 14 | 92/100 | 14.28571429 | <0.0001 |
|  |  |  |  |  |  |
| Fig 5D | N2, DMSO,NAC | 17 | 114/120 | -- | -- |
|  | N2, 5mM MTZ, NAC | 17 | 120/120 | -- | 0.4587 |
|  | N2, 5mM 8-Oac, NAC | 15 | 104/120 | -11.76470588 | <0.0001 |
|  | N2, 5mM Rote, NAC | 22 | 120/120 | 22.72727273 | <0.0001 |
|  |  |  |  |  |  |
| Fig 5D | N2, DMSO,NAC | 14 | 94/100 | -- | -- |
|  | N2, 5mM MTZ, NAC | 10 | 95/100 | -28.57142857 | <0.0001 |
|  | N2, 5mM 8-Oac, NAC | 10 | 94/100 | -28.57142857 | <0.0001 |
|  | N2, 5mM Rote, NAC | 16 | 98/100 | 14.28571429 | <0.0001 |
|  |  |  |  |  |  |
| Fig 5D | N2, DMSO,NAC | 14 | 98/100 | -- | -- |
|  | N2, 5mM MTZ, NAC | 12 | 95/100 | -14.28571429 | <0.0001 |
|  | N2, 5mM 8-Oac, NAC | 12 | 93/100 | -14.28571429 | <0.0001 |
|  | N2, 5mM Rote, NAC | 16 | 93/100 | 14.28571429 | <0.0001 |
